# ﻿A new species of *Pseudodendroides* Blair, 1914 (Coleoptera, Pyrochroidae, Pyrochroinae) from China, with a key to the species

**DOI:** 10.3897/zookeys.1228.142968

**Published:** 2025-02-21

**Authors:** Qi Gao, Xin-Mei Yang, Daniel K. Young, Zhao Pan

**Affiliations:** 1 Key Laboratory of Zoological Systematics and Application of Hebei Province, School of Life Sciences, Institute of Life Science and Green Development, Hebei University, 071002, Baoding, Hebei Province, China Hebei University Baoding China; 2 Department of Entomology, College of Plant Protection, Nanjing Agricultural University, 210000, Nanjing, Jiangsu Province, China Nanjing Agricultural University Nanjing China; 3 Department of Entomology, University of Wisconsin, Madison, WI 53706, USA University of Wisconsin Madison United States of America

**Keywords:** China, fire-colored beetle, identification key, new species, *Pseudodendroides*, taxonomy

## Abstract

*Pseudodendroides* Blair, 1914 is distributed in East Asia, with five described species. Currently, a new species, *Pseudodendroidesfrontalis* Gao & Pan, **sp. nov.**, is described and illustrated from Yunnan Province, China. A key to the species of *Pseudodendroides* is provided and the phylogenetic relationships among *Pseudodendroides* and related genera are briefly discussed.

## ﻿Introduction

The pyrochroine *Pseudodendroides* Blair, 1914 was redefined by Young (1999, 2015) and may be distinguished from other pyrochroine genera by the following character combination: compound eyes large; cranial pits (male) well developed and usually paired; antennal scape long and parallel-sided; posterior margin of abdominal sternite VIII widely emarginate and conspicuously concave (male); parameres of male genitalia short and widely separated for approximately half their length (Young 1999). Young (2015) reviewed the taxonomic history of this genus and transferred two species to the genera *Himalapyrochroa* Young, 2004 and *Sinodendroides* Young, 2005. The following year, he transferred *Dendroidesmadurensis* Pic, 1912 from *Pseudodendroides* to the new genus *Pyroghatsiana* (Young 2016). Currently, five extant species of *Pseudodendroides* are known (Young et al. 2020 recorded six species in error) from Japan and southern China (Lewis 1887; Kôno 1935, 1936; Pic 1955; Nakane 1988).

Three of the five *Pseudodendroides* species have been recorded from China up to now: *P..sulcatithorax* Pic, 1955, *Ps.umenoi* (Kôno, 1936), and *P..uraiana* Kôno, 1935. During the examination of specimens from Yunnan Province, we discovered a new species, *Pseudodendroidesfrontalis* Gao & Pan, sp. nov.. The new species is described and illustrated herein, and a key to *Pseudodendroides* species is provided.

## ﻿Material and methods

The type material is deposited in the Museum of Hebei University, Baoding, China [MHBU (MHBUa = material preserved in 95% ethyl alcohol)]. The specimens were studied using a Nikon SMZ1500 stereomicroscope, and the images were taken using a Canon EOS 5D Mark III (Canon Inc., Tokyo, Japan) with a Laowa FF 100 mm F2.8 CA-Dreamer Macro 2× or Laowa FF 25 mm F2.8 Ultra Macro 2.5–5× (Anhui Changgeng Optics Technology Co., Ltd, Hefei, China). Figures of the antennae were hand drawn using the Nikon SMZ1500 with a camera lucida. Label data are presented verbatim. Line breaks on labels are denoted by a single slash (/).

Most of the terms in the description are from previous literature (e.g., Young 1975). The ocular index (OI) = 100× minimum dorsal distance between compound eyes / maximal dorsal width across compound eyes (Campbell and Marshall 1964).

## ﻿Results

### ﻿Genus Pseudodendroides

Taxon classificationAnimaliaColeopteraPyrochroidae

Blair, 1914

B21F75CA-C6AC-57C2-AEC7-F1938917B8DC

Pseudodendroides Blair, 1914: 314; Young 1999: 1; 2015: 195; Pollock and Young 2008: 415; Young et al. 2020: 566.

#### Type species.

*Dendroidesniponensis* Lewis, 1887, by original designation.

### ﻿Key to the species of *Pseudodendroides*

**Table:** 

1	Male: eyes larger, OI usually less than 25 (Fig. 2), except *P..amamiana* and *Ps.umenoi* (figs 15–16 in Nakane 1988), rami of antennal flagellomeres longer (Fig. 4), posterior margins of abdominal sternites VII and VIII mesally emarginate (fig. 4 in Young 1999)	**2**
–	Female: eyes smaller, OI greater than 30 (Fig. 3), rami of antennal flagellomeres shorter (Fig. 5), posterior margins of abdominal sternites VII and VIII almost straight	**7**
2	Frons with one sub-rounded shallow depression (Fig. 2); pronotum orange-yellow; China: Yunnan	***Ps.frontalis* Gao & Pan, sp. nov.**
–	Frons with cranial pits at least partially divided mesally by well-developed carina; pronotum black	**3**
3	Compound eyes large, OI less than 25; cranial pits paired, with at most a feeble additional transverse ridge	**4**
–	Compound eyes relatively smaller than above, OI greater than 30 (fig. 8 in Young 1999); frons with two distinct cranial pits and a transverse ridge, making pits nearly four chambered	**6**
4	Cranial pits deep, bordered posteriorly by slightly elevated rim, rim slightly emarginate mesally; China: Sichuan	***P..sulcatithorax* (Pic, 1955)**
–	Cranial pits broad, shallow, their posterior margin not elevated	**5**
5	Cranial pits separated posteriorly by mesal carina, pits confluent anteriorly; Japan	***P..niponensis* (Lewis, 1887)**
–	Cranial pits completely separated by mesal carina; China: Taiwan	***P..uraiana* (Kôno, 1935)**
6	Cranial pits subquadrate (fig. 15 in Nakane 1988); Japan: Amami-Oshima	***P..amamiana* (Nakane, 1988)**
–	Cranial pits, especially posterior pits, ovate-rounded; China: Taiwan	***Ps.umenoi* (Kôno, 1936)**
7	Pronotum orange-yellow; flagellomere I without ramus; China: Yunnan	***Ps.frontalis* Gao & Pan, sp. nov.**
–	Pronotum black, at most with red-orange margin; flagellomere I with short ramus	**8**
8	Compound eyes large, OI less than 40	**9**
–	Compound eyes relatively smaller than above, OI greater than 45	**11**
9	Flagellomere I with dorsal face, including ramus flat to slightly concave, foliaceous; China: Sichuan	***P..sulcatithorax* (Pic, 1955)**
–	Flagellomere I sub-cylindrical	**10**
10	Frons between antennal insertions very sparsely punctate; Japan	***P..niponensis* (Lewis, 1887)**
–	Frons between antennal insertions moderately sparsely punctate; China: Taiwan	***P..uraiana* (Kôno, 1935)**
11	Frons densely covered with moderately long, golden setae; Japan: Amami-Oshima	***P..amamiana* (Nakane, 1988)**
–	Frons moderately sparsely covered with short, mostly decumbent setae, especially between compound eyes; China: Taiwan	***Ps.umenoi* Kôno, 1936**

### ﻿ Pseudodendroides frontalis

Taxon classificationAnimaliaColeopteraPyrochroidae

Gao & Pan sp. nov.

6FCCDCDD-1382-5E67-9B5B-26FA4C484983

https://zoobank.org/D522CD7B-C7F2-4721-BD19-8EBC31B7F2DC

Figs 1–10

#### Type locality.

China: Yunnan, Nujiang Lisu Autonomous Prefecture, Lanping Bai and Pumi Autonomous County, Zhongpai Township, Biyuhe Village.

#### Type specimens.

***Holotype***: • ♂, labeled “2023.V / 云南兰坪中排乡碧玉河村 [China, Yunnan, Lanping, Zhongpai Township, Biyuhe Village / elev. 2600–2900 m / 河北大学博物馆 [Museum of Hebei University]”, “HOLOTYPE / *Pseudodendroidesfrontalis* sp. nov. / Det. Gao & Pan” (MHBU). ***Paratypes***: • 1 ♂ 1 ♀, with same label as the holotype (MHBU); 6 ♂♂, labeled “2023.VI / 云南兰坪中排乡碧玉河村 [China, Yunnan, Lanping, Zhongpai Township, Biyuhe Village / elev. 2700–3000 m / 河北大学博物馆 [Museum of Hebei University]”, 5 alcohol specimens additionally with a label of Sample ID for each: “P2J9, P2J10, P3B2, P3B3, P3B4” (1 MHBU, 5 MHBUa); • 1 ♀, “2023.X / 云南怒江福贡石月亮乡 [China, Yunnan, Nujiang, Fugong, Shiyueliang Township / elev. 2500–2700 m / 河北大学博物馆 [Museum of Hebei University]” (MHBU). All paratypes with the label “PARATYPE / *Pseudodendroidesfrontalis* sp. nov. / Det. Gao & Pan”.

#### Diagnosis.

This new species can be easily distinguished from other *Pseudodendroides* species by its orange-yellow pronotum (entirely to largely black in other species). Additionally, males of *Ps.frontalis* lack distinct cranial pits, exhibiting only a shallowly depressed region between the antennal insertions, and the compound eyes are very large, similar to those of *P..niponensis* (OI = 17.4–18.5). By contrast, the frons of other species possesses two distinct cranial pits. The compound eyes of *Ps.umenoi* and *P..amamiana* are relatively smaller than those of *Ps.frontalis* (OI ≥ 30). The females of all *Pseudodendroides* species are morphologically similar. However, the female of the new species differs from those of the other species in the shape of flagellomere I, which is almost without any ramus; there is at most a slight apical prominence in *Ps.frontalis*, with a long or short ramus in the other species.

**Figures 1–10.**
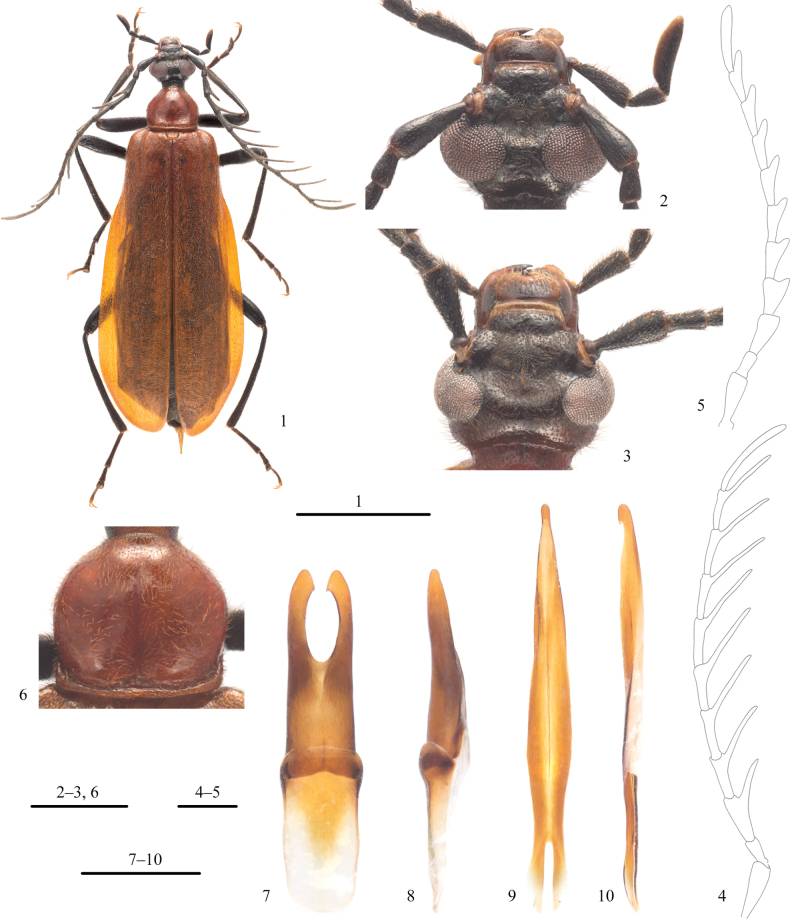
*Pseudodendroidesfrontalis* Gao & Pan, sp. nov., paratypes **1** habitus, male, dorsal view **2, 3** head, dorsal view: **2** male **3** female **4, 5** antenna: **4** male **5** female **6** pronotum, male, dorsal view **7, 8** tegmen: **7** dorsal view **8** lateral view **9, 10** penis: **9** dorsal view **10** lateral view. Scale bars: 5 mm (**1**); 1 mm (**2–10**).

#### Description.

Body length: 16.3–16.8 mm; humeral width: 3.2–3.8 mm.

**Male**: Body (Fig. 1) black, except pronotum, prosternum, and elytra orange-yellow; apex of clypeus, labrum, and mandibles yellow-brown to dark brown. Body covered with medium-length yellow-brown setae; those of antennae and legs short (except coxae, trochanters, and ventral sides of femora); setation sparse on head and pronotum, dense on elytra, and very dense at basal half of depression on frons.

Head (Fig. 2) widest at level of eyes, with irregular small punctures, diameter of punctures less than spacing of punctures; punctures on vertex each bearing a very fine, long seta. Compound eyes large, dorsal distance between eyes narrow (OI = 17.4–18.5). Clypeus and labrum flattened; labrum with anterior margin slightly emarginate. Frons with one sub-rounded large depression between antennal insertions; occiput sharply, transversely concave; genae reduced, short. Antennae (Fig. 4) long, extending back to near middle of elytra; scape slightly and gradually widened apically; pedicel short, approximately half length of scape; flagellomere I shortest; flagellomeres II–IV subequal in length, approximately as long as scape; rami of flagellomeres sub-cylindrical, ramus of flagellomere I shortest, approximately half as long as flagellomere I, rami of flagellomeres I–IV with gradually increasing lengths, rami of flagellomeres IV–VIII subequal in length and shorter than flagellomere IX.

Pronotum (Fig. 6) widest in anterior 2/5, slightly and gradually narrowed posteriad and sharply narrowed anteriad; approximately as wide as head, 0.80–0.90× as long as wide; disc shining, sparsely covered with very small punctures, diameter of punctures distinctly less than spacing of punctures; disc with a shallow, longitudinal mesal groove, a shallow depression at center of base, and a transverse groove along posterior margin. Scutellar shield rounded apically, densely and finely punctate. Legs slender; prothoracic tarsomere V longest, I second longest, II–IV gradually shorter; mesothoracic tarsomere V subequal in length to I, II–IV gradually shorter; metathoracic tarsomere I longest, IV second longest, II–III gradually shorter. Pretarsal claws simple.

Posterior margins of abdominal sternites III–VI subparallel, VII–VIII with posterior margin acutely emarginate mesally. Parameres distinctly longer than phallobase (Figs 7, 8), basal half of parameres fused, each lateral lobe of parameres with a small, subapical tooth on inner margin, in dorsal view (Fig. 7). Penis elongate, somewhat dorsoventrally flattened, gradually narrowed apically, apex nodular-shaped with a small recurved hook (Figs 9, 10).

**Female**: Similar to male, except as follows: depression on frons shallower than that of male and without dense setae inside; compound eyes relatively small (OI = 34.7–42.9), with wider range than male; genae slightly prominent (Fig. 3); antennal rami distinctly shorter than those of male, flagellomere I almost without ramus, only slightly prominent apically (Fig. 5); pronotum slightly wider than that of male, aspect ratio ca. 0.82; posterior margin of abdominal sternite VII straight, that of sternite VIII slightly convex mesally.

#### Etymology.

The specific name comes from the Latin adjective “*frontalis*” meaning “frontal”, in reference to the unique characteristic of the frons of this species in the male, bearing a transverse shallow depression instead of paired cranial pits.

#### Distribution.

China: Yunnan.

## ﻿Discussion

According to the redefinition of *Pseudodendroides* (Young 1999), the male head has paired cranial pits on the frons. However, the frons of the new species, *Ps.frontalis*, is only shallowly depressed. We propose that this new species belongs to the genus *Pseudodendroides* because it fits most of the diagnostic characters of the genus, e.g., the shape of the antennal scape, the abdominal sternite VIII, and the parameres of male genitalia. Thus, *Pseudodendroides* may not be distinguished from other genera based on the shape of the frons in males.

As mentioned in previous literature (Gao et al. 2024a, b), Pyrochroinae is currently known to consist of 15 genera worldwide, but their phylogenetic relationships remain unclear. A preliminary phylogenetic analysis of the subfamily Pyrochroinae based on mtDNA *COI* barcode sequences found that *Pseudodendroides* has very close relationships with the genera *Himalapyrochroa* and *Phyllocladus* Blair, 1914 (Gao, unpubl. Masters thesis). Unfortunately, the relationships among these three genera have still not been resolved on this basis or by results based on morphological comparisons (also see Young 2004, 2013, 2015; Gao et al. 2023).

These three genera share the following morphological characters: the male genitalia are similar, with the fused portions of the parameres being relatively short (approximately half of the total length); the penis is nodular-shaped apically; the antennal scape is elongate and slightly clavate, while the pedicel is elongate and sub-cylindrical. For differences, *Phyllocladus* may generally be distinguished from the other two by the flattened rami of its flagellomeres (versus cylindrical in *Himalapyrochroa* and *Pseudodendroides*). *Pseudodendroides* and *Phyllocladus* share a putative apomorphy associated with the abdominal sternite VIII: the apical margin is widely emarginate and conspicuously concave. However, it is interesting that the cranial pits and the size of the compound eyes in the male are polymorphic in *Phyllocladus* and *Pseudodendroides*. The frons of *Ps.frontalis* n. sp. and all species of *Himalapyrochroa* lacks distinct cranial pits, bearing only a shallow depression (distinct paired cranial pits in other *Pseudodendroides* species and all species of *Phyllocladus*). *Phyllocladusgrandipennis* (Pic, 1906), *Ps.frontalis* n. sp., and *P..niponensis* have very large eyes (the minimum dorsal distance between the eyes is distinctly less than the transverse width of each eye); whereas the other species of both *Phyllocladus* and *Pseudodendroides* have smaller eyes (the minimum dorsal distance between the eyes is at most as wide as the transverse width of each eye), which is shared with *Himalapyrochroa* species.

## Supplementary Material

XML Treatment for Pseudodendroides

XML Treatment for Pseudodendroides frontalis

## ﻿Additional information

### Author ORCIDs

Qi Gao https://orcid.org/0000-0002-5902-9276

Xin-Mei Yang https://orcid.org/0009-0008-4480-7371

Daniel K. Young https://orcid.org/0000-0002-5711-2519

Zhao Pan https://orcid.org/0000-0001-7798-0009

### Data availability

All of the data that support the findings of this study are available in the main text.
